# SH3 Domain-Peptide Binding Energy Calculations Based on Structural Ensemble and Multiple Peptide Templates

**DOI:** 10.1371/journal.pone.0012654

**Published:** 2010-09-15

**Authors:** Seungpyo Hong, Taesu Chung, Dongsup Kim

**Affiliations:** Department of Bio and Brain Engineering, KAIST, Daejeon, South Korea; Center for Genomic Regulation, Spain

## Abstract

SH3 domains mediate signal transduction by recognizing short peptides. Understanding of the driving forces in peptide recognitions will help us to predict the binding specificity of the domain-peptide recognition and to understand the molecular interaction networks of cells. However, accurate calculation of the binding energy is a tough challenge. In this study, we propose three ideas for improving our ability to predict the binding energy between SH3 domains and peptides: (1) utilizing the structural ensembles sampled from a molecular dynamics simulation trajectory, (2) utilizing multiple peptide templates, and (3) optimizing the sequence-structure mapping. We tested these three ideas on ten previously studied SH3 domains for which SPOT analysis data were available. The results indicate that calculating binding energy using the structural ensemble was most effective, clearly increasing the prediction accuracy, while the second and third ideas tended to give better binding energy predictions. We applied our method to the five SH3 targets in DREAM4 Challenge and selected the best performing method.

## Introduction

Peptide recognition domains (PRDs) recognize peptides and relay the signals. There are diverse PRDs that are involved in diverse signal transduction pathways. In many cases, they work in a modular fashion [1], [2], which implies that the interaction between peptides and PRDs can be dealt with separately. Due to their importance in cellular signal transduction, numerous experimental and computational studies have been performed to characterize their binding specificity. Development of the SPOT synthesis method has contributed significantly to our understanding of the binding specificity of PRDs. It has allowed measurement of the binding affinity of PRDs to multiple peptide sequences [3]. High-throughput analyses have been performed using this technique [4], [5]. Moreover, the accumulation of data has made it feasible to develop diverse computational methods for the prediction of PRD-peptide interactions. A molecular dynamics (MD) simulation technique was used to study interactions between SH2 domains and the peptides [6]. The global motion of the domains was studied using a Gaussian Network Model to explain the promiscuity of the PDZ domain [7]. Position specific scoring matrices were constructed to capture the interaction characteristics of SH3 domain families [8]. A 3D QSAR method was used to predict the affinity of peptides on MHC [9]. Physical energy terms, such as van der Waals, electrostatic, and desolvation energies, were used to predict the energy of domain peptide interactions [10]. With these physical energy terms as feature vectors, machine learning techniques such as using a support vector machine were applied to peptide-SH3 domain interaction prediction [11], [12]. Statistical energy derived from the binding energy data and the complex structures was used to describe the specificity of SH2 domain [13]. In other studies, protein-protein interaction data such as yeast two-hybrid was used to generate probabilistic models that can predict peptide sequences binding to SH3 domains [14], [15].

In DREAM4 (URL: http://wiki.c2b2.columbia.edu/dream/index.php/D4c1), prediction methods for peptide-PRD were assessed by blind test for three types of peptide recognition domains: kinase, PDZ, and SH3. The development of prediction methods is needed because even current high-throughput techniques, such as SPOT, are limited to rather small sequence variations [16]. Computational predictions for peptide-PRD can be applied to far more diverse peptides. Experimentally confirmed pre-publication binding data were kindly provided by Sachdev Sidhu at Terrence Donnelly Center for Cellular and Biomolecular Research, University of Toronto and Ben Turk at Department of Pharmacology, Yale University. The goal of the project was to predict the position weighted matrix (PWM), which defines the binding specificity of the target domains. Only the sequences of domains were given. We participated in the SH3 domain peptide specificity prediction category, for which five domains were given.

For SH3 domain binding peptides, two canonical motifs are frequently observed: +xxPxxP (class I motif) and PxxP x+ (class II motif), where ‘x’ denotes any of 20 amino acids, ‘+’ positively charged amino acids, and ‘P’ proline. These peptides are restricted to triangular prism shape conformation due to the ‘PxxP’ motif [17], [18]. In this conformation, side chains of neighboring amino acids are directing to other directions. In this way, each amino acid of the peptide can be assumed to have little interaction with the inter-peptide amino acids so that each position can be considered to be independent of each other, which is a necessary property for representing the binding specificity with the PWM.

We approached this challenge by modeling the domain-peptide complex structures and then calculating the energy matrices describing the binding energy contribution of a specific amino acid at a specific position, excluding any prior experimental binding energy information. Fernandez-Ballester et al. tried a similar approach [19]. In their research on the SH3 domain, they particularly focused on the generation of good template structures. However, peptides can have many conformations due to their thermal fluctuation. Thus, explicit consideration of structural ensemble would improve the binding energy prediction. In other previous researches, positions of peptides binding on PRD domains were considered as being fixed at the canonical motif. However, for general sequences without canonical motifs there is no clue how such methods can be applied. Furthermore, there is no guarantee that peptides in canonical motifs have the lowest energy. Thus it is worthwhile to test the effect of different sequence-structure mapping; in other words, the lowest energy binding position of peptide-domain needs to be investigated.

In this study, we developed three ideas: utilizing the structural ensemble sampled from a molecular dynamics simulation trajectory, using multiple peptide templates, and optimizing the sequence-structure mapping. We validated our ideas using Landgraf et al.'s data set for SH3 domain-peptide specificity [5]. Using this method, we predicted the binding specificity of 5 DREAM4 SH3 domain targets.

## Results

**Figure 1:**
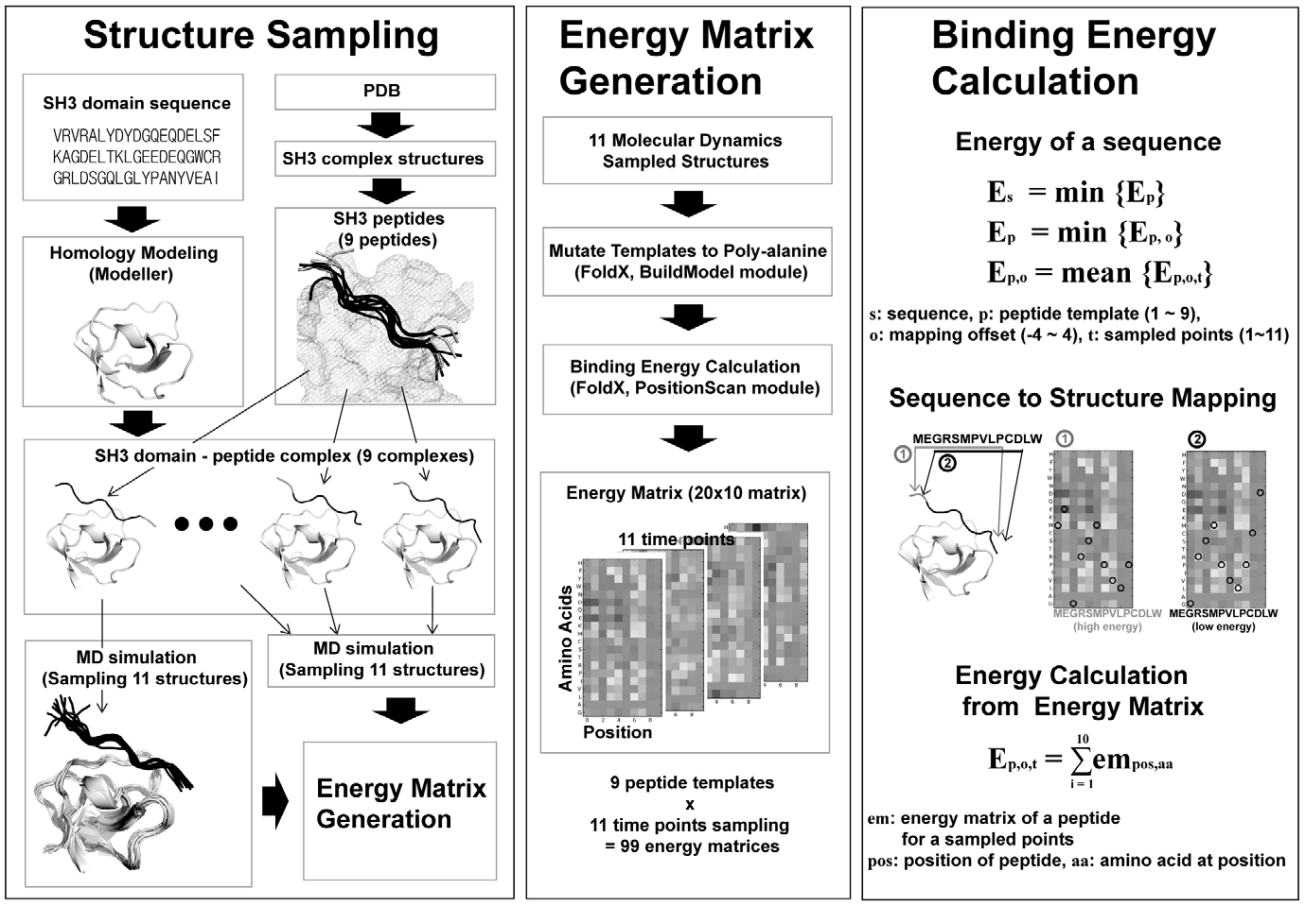
Ensemble Based Binding Energy Calculation Method. Our method is composed of three steps: structure sampling, energy matrix generation, and binding energy calculation. Initial complex structures were generated by superimposing the peptides of crystal structures to the modeled SH3 domains. For each initial complex the near binding state conformations were sampled by molecular dynamics simulation. Sampled structures were used in calculating the contribution of each amino acid on the binding energy on each position, which is converted into energy matrices. The resulting energy matrices were used to calculate the binding energy of peptides.

We tested the validity of the three ideas which we expected would improve our ability to predict the binding energy between SH3 domains and peptides: (1) utilizing the structural ensembles sampled from a MD simulation trajectory, (2) using multiple peptide templates and (3) optimizing the sequence-structure mapping. We tested the validity of these ideas on ten previously studied SH3 domains (ABP1, BOI1, BOI2, LSB3, MYO5, RVS167, SHO1, YSC84, Amphyphisin, Endophilin) for which SPOT analysis data were available [5]. The BLU values in the SPOT data were converted into energies by taking a logarithm, assuming that the number of domain-peptide complexes is proportional to BLU value. The overall scheme of the method is shown in Figure 1. For each SH3 domain, 9 peptide structures collected from the PDB were used as a template to construct the SH3 domain-peptide complexes. For each SH3 domain-peptide complex structure, a structural ensemble composed of 11 different conformations sampled from the MD simulation trajectory was generated. As a result, a total of 99 (9 peptide structures ×11 time points) complex structures were generated for each domain, and those complex structures were used as templates to make 99 energy matrices using FoldX [20], [21]. For each target peptide, the binding energy was calculated using those 99 energy matrices.

### Effect of utilizing the structural ensemble sampled from MD simulation trajectories

We tested whether utilizing the structural ensemble sampled from MD simulation trajectories would have any advantage over using a single complex structure. To test this, among nine different domain-peptide complex models we selected a single structure that had the highest correlation with SPOT data (the best complex model). Using this structure model and optimizing the sequence-structure mapping, we calculated the correlation coefficients for the following three different cases: (i) using a single conformation sampled at a single time point, (ii) using the single best conformation among 11 different conformations for each SH3 domain, and (iii) using the structural ensemble sampled at 11 different MD simulation time points. The results are shown in Table 1.

**Table 1:** Effect of structural ensemble sampled from MD simulation trajectory.

SH3 Domain	Single Conformation*	Best Conformation	Multiple Conformations
**ABP1**	0.32±0.03	0.37	**0.39**
**Amphyphisin**	0.33±0.13	**0.53**	0.43
**Endophilin**	0.41±0.06	0.48	**0.53**
**MYO5**	0.23±0.10	0.33	**0.36**
**RVS167**	0.31±0.07	**0.44**	0.38
**SHO1**	0.35±0.05	0.42	**0.43**
**LSB3**	0.57±0.06	0.65	0.65
**YSC84**	0.34±0.16	**0.55**	0.47

The Pearson's correlation coefficients between the predicted binding energies and SPOT data are shown.

*Average correlation coefficient of 11 conformations.

It should be noted that the 11 conformations sampled from the MD trajectory are all equivalent in the sense that there is no clear way to tell which conformation is the most appropriate for calculating the binding energy for a given peptide. Therefore, in order to estimate the prediction performance for case (i), we calculated the Pearson's correlation coefficient using each conformation separately for the 11 sampled MD conformations and then calculated the average correlation coefficient. For case (ii), to select the best conformation, one with the highest correlation was chosen. In some cases, such as ABP1, the difference between the average performance and the best performance was small. However, in many cases, such as Amphyphisin and YSC84, the difference was rather large, indicating that the binding energy calculated using FoldX can be sensitive to a small structural variation. The results indicate that choosing a good structural template is critical for predicting the binding energy accurately.

In the fourth column of Table 1, the correlation coefficients for case (iii) are shown. It is clear that using multiple conformations sampled from MD simulations always improves the prediction accuracy compared to the case where only one conformation was used for the prediction. Overall, from Table 1, it is evident that case (ii) and case (iii) produced comparable prediction accuracy. Notably, in four domains (ABP1, Endophilin, MYO5, SHO1), the prediction accuracies were even better than that of the single best conformation case. Considering the fact that choosing the “best” template structure for any given peptide *a priori* is impossible in most cases, the results suggest that utilizing multiple conformations generated by MD simulations is the best strategy.

**Figure 2:**
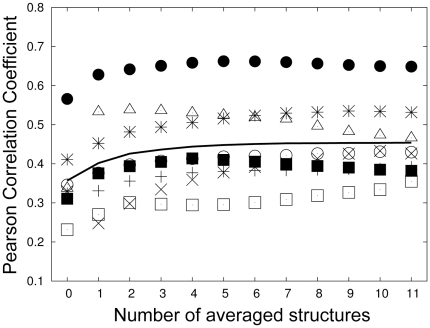
Performance Dependency on Number of Averaged Energies. Out of 11 conformations sampled via molecular dynamics simulation, the average energy of *n* lowest energies was used as the binding energy. At *n* = 0, the average performance when a single conformation was used for calculation is plotted. **‘+’**: ABP1, **‘×’**: Amphyphisin, ‘*’: Endophilin, empty box: MYO5, filled box: RVS167, empty circle: SHO1, filled circle: LSB3, triangle: YSC84, line: averaged performance.

We further analyzed how prediction accuracy varied according to the number of conformations used in case (iii). To do this, we calculated the binding energies using only the *n* lowest energy conformations (*n* = 1, …,11) for each peptide. We chose the conformations in this way because we wanted to test whether simply choosing the single lowest energy conformation for each peptide is a better strategy than choosing multiple conformations. In Figure 2, we also showed the correlation coefficients for the case-(i) (*n* = 0). If we compare the points at *n* = 0 and *n* = 1, it is clear that choosing the conformation with the single lowest energy for each peptide was always better than choosing a single arbitrary conformation. The results shown in Figure 2 also show that as the number of conformations increased, the prediction accuracy generally increased, indicating that choosing multiple conformations is better than choosing one or two lowest energy conformations.

### Effect of Using Multiple Peptide Templates

Our second idea is nearly identical to that of recent study of Fernandez-Ballester et al. [19] in which they used FoldX for binding energy calculations and chose a single template among multiple peptide templates based on the minimum energy criteria. However, as we used structural ensembles and optimization of sequence-structure mapping for the binding energy calculation, we tested this idea again in combination with two other new methods. Similar to conformational selection from 11 sampled MD conformations, there is no clue to which peptide is most suitable for a given sequence. When a sequence has one of the two canonical motifs, this information can be used. In Table 2, we compared the case where the peptide is chosen by the energy and the case where the peptide is chosen by the type of canonical motif in the sequence. Sequences in the SPOT data for RVS167, SHO1, LBS3, and YSC84 have distinct class I and II motifs. Considering the motifs separately, energy based peptide selection is better in 6 out of 8 cases. Thus we can conclude that choosing the peptide template that has the lowest binding energy is a reasonable strategy.

**Table 2:** Effect of using multiple peptide templates.

	Energy Based Selection	Best Peptide Template		Average†	
Domain*			Peptide Template†	Class I	Class II
**ABP1**	0.**42**	0.39	4 (II)	0.36	0.32
**Amphyphisin**	0.20	0.43	8 (II)	0.03	**0.35**
**Endophilin**	**0.49**	0.53	9 (II)	0.30	0.41
**MYO5**	**0.21**	0.36	2 (I)	0.20	0.17
**RVS167**	**0.35**	0.38	7 (II)	0.25	0.30
**RVS167(I)**	0.29	0.48	6 (II)	0.26	**0.36**
**RVS167(II)**	**0.41**	0.48	7 (II)	0.26	0.36
**SHO1**	**0.43**	0.43	3 (I)	0.36	0.32
**SHO1(I)**	**0.49**	0.52	3 (I)	0.45	0.30
**SHO1(II)**	0.23	0.26	2 (I)	**0.25**	0.21
**LSB3**	**0.68**	0.65	9 (II)	0.19	0.59
**LSB3(I)**	**0.56**	0.47	4 (II)	0.39	0.42
**LSB3(II)**	**0.72**	0.71	9 (II)	0.35	0.66
**YSC84**	**0.46**	0.52	9 (II)	0.07	0.46
**YSC84(I)**	**0.41**	0.40	3 (I)	0.26	0.25
**YSC84(II)**	**0.52**	0.57	9 (II)	0.19	0.49

The Pearson's correlation coefficients between the predicted binding energies and SPOT data are shown.

*When sequences are separated into Class I and Class II, the class is marked in parentheses. Class I has (R/K)xxPxxP motif and Class II has PxxPx(R/K) motif. ABP1, Amphyphisin, Endophilin, and MYO5 do not have the canonical SH3 motifs.

† Peptides 1, 2, and 3 have Class I orientation, and peptides 4, 5, 6, 7, 8, and 9 have Class II orientation. Class I and Class II are marked in parentheses.

There was a peptide template that had better performance than the peptide selected by the lowest energy criteria. However, there is little information on which peptide template would be most suitable for a particular SH3 domain. Thus, using multiple peptide templates and selecting the binding peptide based on the lowest energy criteria would be the best approach unless there is some prior information about the best template peptide.

### Effect of Sequence-Structure Mapping

SPOT data contains information from which the binding affinity of a peptide to a certain SH3 domain can be obtained, but it does not provide information on how the peptide binds to the SH3 domain. In previous studies, all peptides were assumed to bind to the SH3 binding pocket in the canonical binding mode. However, inspection of PDB structures revealed that in some cases peptides resided in the binding pocket one residue off from the canonical binding mode. This inspired us to analyze the different sequence- structure mappings other than the canonical binding modes. In addition, the blind test in the DREAM4 project motivated us to develop a method which was not dependent on the canonical binding motifs.

Sequences in the SPOT data can be mapped on a peptide structure in several ways. We selected mapping with the lowest energy as the optimal alignment. We compared this energy with that of the canonical motif mapping. To exclude the peptide selection effect, we used the peptide template with the best performance. The ensemble structure concept was used in all calculations. For 8 out of 12 cases, the optimization of sequence-to-structure mapping increased the prediction performance, as shown in Table 3. The encouraging result was that deteriorating performances in the remaining 4 cases was very small. These results indicate that the sequence-structure mapping clearly improves the binding affinity prediction.

**Table 3:** Effect of sequence-structure mapping.

Domain†	Alignment(best peptide)*	Without alignment (best peptide)**
**ABP1**	**0.39**	0.36 (−3, II)
**Amphyphisin**	0.43	**0.47** (−1, II)
**Endophilin**	0.53	**0.54** (−1, II)
**MYO5**	**0.36**	0.31 (−3, I)
**RVS167(I)**	**0.48** (0.25) ‡	0.34 (−3, I)‡
**RVS167(II)**	0.48	**0.48** (0, II)
**SHO1(I)**	**0.52**	0.50 (−3, I)
**SHO1(II)**	**0.26** (0.24) ‡	0.24 (0, II)‡
**LSB3(I)**	**0.56** (0.44)‡	0.43 (−3, I)‡
**LSB3(II)**	**0.71**	0.69 (0, II)
**YSC84(I)**	**0.40**	0.38 (−3, I)
**YSC84(II)**	0.57	0.57 (0, II)

The Pearson's correlation coefficients between the predicted binding energies and SPOT data are shown.

† When sequences are separated into Class I and Class II, the class is marked in parentheses. Abp1, Amphyphisin, Endophilin, and Myo5 do not have the canonical SH3 motifs.

*Pearson's correlation coefficient for the best peptide template when alignments are adjusted.

**Pearson's correlation coefficient for the best template peptide when the alignment is fixed to that of canonical motif PxxP. The offset and class of peptide templates are indicated in parentheses.

‡ Cases when the class of the best peptide template is inconsistent with the class of sequence motifs. The best peptide belonging to the sequence motif is indicated in parentheses in the second column. The correlation of fixed alignment for that peptide is shown in the third column.

### Comparison to Previous Methods

The three ideas proposed in this study turned out to improve the binding energy calculation. For further evaluation, we compared our results to those of other prediction methods by Fernandez-Ballester et al. [19] and by Hou et al. [11]. The area under the receiver-operating curve (AROC) was calculated for the classification of binder and non-binder. We used the method of Fernandez-Ballester et al. for a comparison: The top 100 and bottom 100 binding energy sequences were used as binders and non-binders, and the AROC values in their publication were compared to those of our values. As shown in Table 4, our method was better than the previous method for four out of six domains. For the comparison to the method of Hou et al., binder and non-binder data in Hou's publication were used. Our method was better than the method of Hou et al. for four out of five domains.

**Table 4:** Comparison to Other Binding Energy Calculation Methods.

SH3 domain	Fernandez-Ballester*	Our Method	Hou*	Our Method
ABP1	0.83	**0.88**	–	–
BOI1	**0.67**	0.55	**0.84**	0.72
LSB3	0.96	**1.00**	0.91	**0.95**
MYO5	**0.88**	0.74	0.59	**0.66**
RVS167	0.70	**0.88**	0.78	**0.86**
SHO1	0.83	**0.87**	–	–
YSC84	–	–	0.89	**0.96**

Area under ROC curves (AROC) are shown.

*Methods by Fernandez-Ballester [19] and Hou used different data sets[11]. Accordingly, our method was compared with the two methods separately.

### DREAM4 targets

The same analysis was performed for the DREAM4 Peptide Recognition Domain Specificity Prediction Challenge for SH3. We applied our method on the 5 DREAM4 target domains with all three ideas discussed above. Of 5 domains provided for challenges, 3 gold standard PWM matrices were revealed (homology to FISH, Intersectin-1-5 and PACSIN1), and the results for those three domains are discussed here.

**Figure 3:**
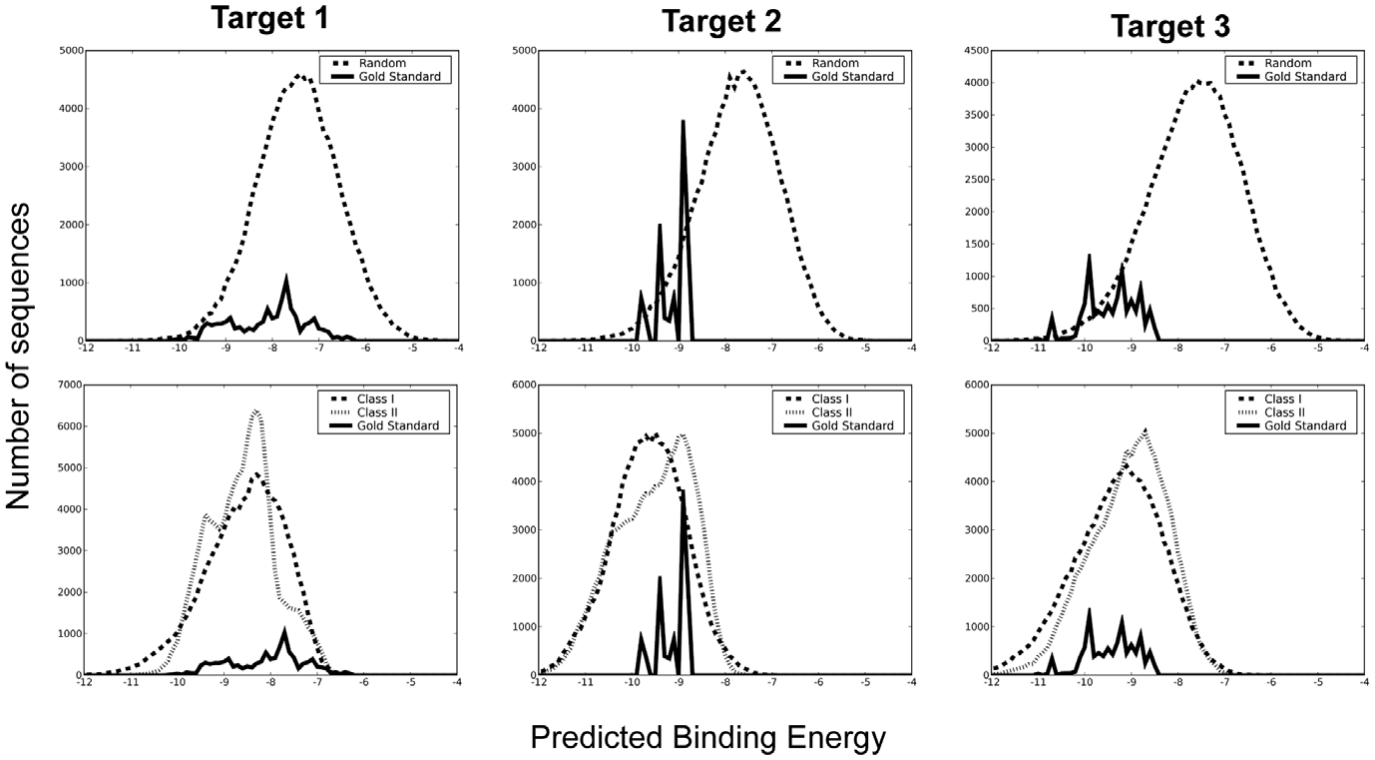
Distribution of Predicted Binding Energy for DREAM4 Target. Binding energies were calculated for randomly generated sequences (upper panel, dashed lines), for random sequences with canonical SH3 binding peptide motifs (lower panel, dashed lines), and for sequences derived from the DREAM4 Gold Standard (solid lines).

For each DREAM4 domain, the binding energies were calculated for three sequence groups: random sequences, random sequences with PxxPxR and RxxPxxP motifs, and sequences generated from the DREAM4 gold standard PSFM. The distributions of the predicted binding energies for each group are shown in Figure 3. For the second and third DREAM4 targets, our method was able to distinguish the DREAM4 gold standard from random sequences, as shown in the upper panel of Figure 3. However, our method failed to characterize the first DREAM4 target.

As the binding motifs were known for SH3 domains, comparison with the sequences with these motifs is a more stringent way to validate our method. In this case the enrichment of the gold standard sequences in the lower energy region was observed for the third target (lower panel of Figure 3).

**Figure 4:**
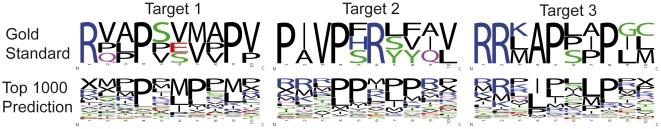
DREAM4 Gold Standard and Predicted PSFM. Position specific frequency matrices are represented with WebLogo [22]. Gold Standards are disclosed for three targets out of five challenges. They are displayed on the upper panel. The PSFM of 1000 sequences with 1000 lowest energies are displayed on the lower panel. Target 1: Homology to FISH, Target 2: Intersection-1-5, Target 3: PACSIN1. In case of target 2, the first position of the DREAM4 fold standard is matched with the fourth position in our prediction.

To further compare the predicted results, the position specific frequency matrix (PSFM) was compared using WebLogo [22] (Figure 4). Among 10^5^ randomly generated sequences, the sequences for the 1000 lowest energies were collected and amino acids frequencies at each position were counted to generate the PSFM. In some sequence-structure mapping, some positions may not be assigned to any amino acid. For those cases, the letter ‘X’ was used to represent them. For the second target, there was some similarity between the N-terminal region of the gold standard and the middle region of our results. However, as the canonical motif, PxxP, lies at the N-terminal region, our structural model could not fully cover the regions constituting the gold standard. In target 3, the predicted PSFM showed some pattern matching with the gold standard (Figure 4). The positions of positively charged and hydrophobic amino acids in the predicted sequences matched well with those in the DREAM4 gold standards.

## Discussion

The overall results indicate that binding energy calculation utilizing structural ensembles sampled from MD simulation trajectories clearly increased the accuracy of binding energy calculations, while using multiple peptide templates tended to give better binding energy predictions. In addition, sequence-structure mapping appeared to improve the prediction accuracy. To test our three ideas more rigorously, we need to test each idea separately in various settings, in addition to the testing that we performed in our work. However, it seems unlikely that the three ideas would work better only for the situations that we set up for the testing.

The improvement of binding energy calculation using ensemble structures might originate from two conditions: the ensemble nature of peptide–protein complex structures and the reduction of computational errors by averaging. In many binding energy calculation methods including FoldX, it is commonly assumed that protein backbone structures are fixed and only a finite number of side chain conformations (rotamers) are considered for calculating the binding energy. The reason for this assumption is purely computational, simply to reduce the computational cost. In reality, however, domain-peptide complexes exist in many different conformational states, dynamically moving from one conformation to another. It is likely that SH3 domain-peptides complexes bind together in various binding modes. Our method captures some of this dynamic nature of proteins. We assumed that MD simulation could produce diverse conformations that could reasonably represent the whole conformational space of domain-peptide complexes. The results seem to validate our hypothesis. Our results are consistent with several recent computational methods based on the ensemble of structures, such as CC/PBSA, in which using the Concoord algorithm diverse structures are sampled to predict the stability or binding affinity change upon mutations [23]. ClusPro considers a cluster of docked structures to predict the real docking conformation [24].

Compared to other methods, our method showed better performance. In the previous binding energy calculations by Fernandez-Ballester et al, SH3-domains structures and peptide-SH3 complex structures were constructed with careful manual inspection and computational structure analysis [19]. For BOI1 and MYO5, Fernandez's work was better than ours. The low sequence homology of the template in BOI1 might be responsible for this. It is unclear why our method failed for MYO5 even though a crystal structure was used. One cause may be the limited number of peptide structures in our method, nine, while Fernandez-Ballester's method used 29 peptides. Though linear independency of interaction was assumed in our method, the backbone structures of the peptides are dependent on the initial sequence of the peptide. Thus, using more diverse peptides might be required to predict binding energy for more diverse sequences. As the method of Hou et al. used different energy function (MM/GBSA) from ours (FoldX), it is not clear whether the performance difference came from energy function or the ideas tested in this study, but it is clear that our method could discriminate binders and non-binders better than the method of Hou et al.

For the two SH3 domains, BOI1 and BOI2, the prediction was almost random. The low sequence homology of the template structures (34% and 33%, respectively) might have caused the failure in the homology modeling. Therefore, application of our methods to the domains whose structures cannot be reliably predicted would be inappropriate.

In DREAM4, the p-value of the PSFM was evaluated in selection of the best performer. The p-value was estimated from the distribution of Frobenius distances between random PSFMs and DREAM4 gold standard PSFM. However, it might not be a good measure for performance evaluation. Simple PSFMs with the perfect matching of the motifs produce significantly small p-values: p-values for PxxPxR motif are 1.4×10^−33^ and 1.0×10^−100^ (effectively zero) for Target 1 and Target 2, and p-value for RxxPxxP motif is 1.0×10^−100^ for Target 3. The estimated p-values of our PSFMs were 2.07×10^−33^, 1.94×10^−20^, and 3.12×10^−31^ for Target 1, Target 2, and Target 3, respectively. Note that the low p-value for Target 1 is somewhat contradictory to the low discrimination power in energy distribution (Figure 3, Target 1). Therefore PSFM based Frobenius distance might not be a good measure for the performance measure. Instead, prediction of binding energy for each peptide would be a better performance measure.

In this study, we developed several methods to improve binding energy calculation and they showed promising results. However, the overall performance was not sufficient to accurately predict the binding specificity of many SH3 domains. For three blind tested SH3 domains in DREAM4, our method could not predict the general pattern of binding peptides in one case. This calls further research on the binding energy calculation. Our method also requires a large number of computations due to the conformation sampling process with MD simulation. Moreover the sampled conformations are highly dependent on the sequences of the peptides. Thus, development of more efficient and general conformation sampling methods would be required to improve computational binding energy prediction.

## Materials and Methods

Overall, our methods are composed of three parts: (1) structure sampling, (2) energy matrix generation, and (3) binding energy calculation (Figure 1). SH3 domain–peptide complex structures were generated by homology modeling, and for each complex structure an ensemble of structures was sampled from the MD simulation trajectories. Using those sampled complex structures as templates, energy matrices were calculated by running FoldX. These matrices contain the binding energy contribution of each amino acid at each position. The binding energy of a given sequence was then calculated with the energy matrices.

### Structure Sampling

#### Homology Modeling

In this work, we studied 15 SH3 domains including 5 DREAM4 targets. The structures of ABP1, Endophilin, MYO5, SHO1 and LSB3 are available in the PDB: 1JO8, 3IQL, 1ZUY, 2VKN and 1OOT, respectively. For the remaining 10 domains we generated their structures by standard homology modeling procedures. The structure with the highest sequence identity to each domain was searched in PDB [25] and was used as the template structure. For DREAM4 targets, 2DNU (50% sequence identity), 1UKL (75%), 2DRK (49%), 1W6X (48%), and 2DBM (56%) were used. For the other 5 domains (Amphyphisin, BOI1, BOI2, RVS167, YSC84), for which SPOT analysis data [5] were available, 1BB9(55%), 2CUC(34%), 2FPD(33%), 1SSH (50%), and 2A08 (97%) were used. Modeller 9v2 with a default option was used for the homology modeling [26] to generate structure models.

#### Collecting Peptide Structures

Thirty SH3 domain-peptide complexes were collected from the PDB. Redundant peptides were removed. Nine structures with relatively high sequence diversity and with linear peptide conformation were chosen and used as peptide templates in generating domain-peptide complex structure models. Three complexes (1ABO, 1JU5, 1QWF) are belonged to class I, and six complexes (1AVZ, 1B07, 1CKA, 1JEG, 1PRM, 1SSH) to class II. For each peptide, at most ten amino acid residues of peptides flanking the SH3 domain center were used. For peptides with less than 10 residues, alanines were added to N- and/or C- terminus using Modeller9v2.

#### Sampling SH3 domain-peptide complex structures

The initial structures of the SH3 domain-peptide complexes were made by structurally aligning the SH3 domains of the complexes onto the target SH3 domains. PyMOL was used for superimposition and inspection of the structures. (DeLano, W.L. The PyMOL Molecular Graphics System. (2008) DeLano Scientific LLC, Palo Alto, CA, USA.) When a clash occurred, the side chains were slightly adjusted to avoid the clash. A Gromacs 4.0.2 package was used for the MD simulation with the Amber03 force field [27], [28], [29]. Initially, energy minimization was performed with the steepest descent method for 5000 steps, followed by 50 ps equilibration with position constraints to the heavy atoms of the complexes. The simulation system was then heated from 100 K to 310 K for the first 500 ps, followed by equilibration up to 2 ns. From the 2 ns to 5 ns trajectories, 11 structures for each SH3-peptide complex structure were sampled with 300 ps uniform intervals.

### Energy Matrix Generation

Similar to the work done by Fernandez-Ballester et al. [19], we assumed that each residue of the peptide independently contributes to the total binding energy. Under this assumption, the peptide binding affinity of each SH3 domain can be expressed as a 20×10 matrix where each element represents the binding energy of a particular amino acid at a specific position of the peptide. To calculate the energy matrices, we used FoldX 3.0b [20], [21]. Specifically, a peptide was mutated into poly-alanine, and then alanine at each position was mutated into one of 19 amino acids and their binding energies were calculated using the PositionScan module in FoldX. This calculation was performed for 10 residue positions of the peptide. The resulting 20 (amino acids) by 10 (positions) matrix was defined as an ‘energy matrix.’ The binding energy of any given sequence can be calculated by looking up the energy matrix and summing up the binding energy contribution from each amino acid at all positions.

### Binding Energy Calculation

In this study we assumed that a peptide-domain binding state was represented by multiple structures, *i.e.,* a structural ensemble. Thus 11 MD sampled conformations represent a binding state, and an average of their energies is regarded as the binding energy of the binding state. To represent diverse binding modes, 9 peptides with different sequences and structures were used. We assumed that a peptide with a certain sequence has one of those binding modes, and that the mode of the peptide with the lowest energy is the closest one to the natural binding mode of the sequence.

In addition, as there is no one-to-one mapping from a sequence to a peptide structure, we considered several different mappings, and one with the lowest energy was chosen.

More specifically, our structural model for a peptide has ten positions, P_1_P_2_…P_10_. The consequent energy matrix has a binding energy corresponding to each amino acid. Then, for a sequence with length 10, S_1_S_2_…S_10_, its energy was calculated from the summation of energies of amino acids for each position. However, a sequence with length *n*, S_1_S_2_…S_n_, can be placed on the position in many different manners, such as (P_1_, S_1_), (P_2_, S_2_), …, (P_10_, S_10_) or (P_1_, S_2_), (P_2_, S_3_), …, (P_10_, S_11_). We denoted the first case as ‘offset 0’ and the second case as ‘offset −1’. Binding energy was calculated using the mapping with offset from −4 to 4.

### Generation of DREAM4 Evaluation Sequences

To evaluate DREAM4 gold standard, 10^4^ sequences were generated using the gold standard PSFM. Random sequences were generated with equal probability for each amino acid. Class I and Class II peptides were generated by fixing RxxPxxP and PxxPxR motifs but varying the other sites.
